# Annotations

**Published:** 1901-12-21

**Authors:** 


### ANNOTATIONS.

### Asylum Treatment and "Medical Relief."

In the High Court, last Monday, judgment was given in a case which involved the question whether a man is made a pauper, and is thus deprived of his vote, by the fact of his wife being maintained at the expense of the rates in a lunatic asylum. This was an appeal from a case stated by the revising barrister for the city of Gloucester. The voter's wife had been maintained for a period of about two years in the County Lunatic Asylum at the expense of the union, and he had not paid anything towards her support. In giving judgment, Mr. Justice Channell said that in all cases in which relief is given which is more or less of the nature both of medical relief and ordinary relief a question of fact arises for the decision of the revising barrister as to the character of the relief, and as to which form of relief is incidental to the other. He said—and this coming from a judge on the bench is important—"Where in consequence of illness (and insanity is undoubtedly illness) a voter or a member of his family whom he is bound to support is given medical relief, it is not the less medical relief within the statute because a certain amount of ordinary sustenance is given as well as medicine. . . . Where a member of a voter's family is attacked with insanity, and is in consequence removed to an asylum at the expense of the union we need have no doubt, and the revising barrister in this case seems to have had no doubt, that the case was in the first instance one of medical relief. But where the insane person (being a person for whose support the voter is liable) remains permanently in the asylum and no payment is made by the voter, we think a question of fact arises as to whether the relief has not become ordinary relief as distinguished from medical." The appeal was dismissed. The points of interest are that "insanity is undoubtedly an illness;" that its treatment in an asylum *may* be properly regarded as medical relief; and the nice distinction which is drawn between the case in which the sustenance is a mere incident of the treatment, and the one in which the medical treatment seems to be thrown in with the support and maintenance.

### Hospital Building "While you Wait."

From the report of the proceedings at the last meeting of the Metropolitan Asylums Board we gather that we are to be again regaled with a *tour de force* in the building line at the expense of the ratepayers. It is not the first time, nor will it be the last. As is well known, the managers always have at hand a well-sounding excuse in the dilatoriness and perversity of their good friends the Local Government Board to explain away their fondness for building in a hurry. But that does not lessen the wastefulness of the proceeding, and when we hear that the following buildings are to be erected in about eight weeks from the giving of the order, namely, isolation accommodation, more provision for assistant medical officers, extensions of the staff bathing accommodation, and extensions of the quarters for nurses, domestics, and other subordinate servants, and that these things, built on the "while you wait" principle, are to cost £16,900, we cannot help wondering how much they would have cost if they had been erected under ordinary conditions. Of course all this is a trifle compared with what happened at Tottenham and Tooting, but as Mr. Scovell is good enough to say that after the recess the committee will come again with "probably a much larger proposal" we are apparently at the beginning of a new series of wasteful performances by "lightning artists" in hospital construction. The Asylums Board managers are doing well for poor, helpless, subdivided London, in providing for its small-pox cases. But they have always been credited with a readiness to "take it out" of the Local Government Board, by indulging in extravagant expenditure, whenever it could be made to appear that they were driven to this course by the obstructive action of the superior body, and in view of the discussions which have from time to time arisen in regard to the Joyce Green Hospital, it is perhaps natural that the managers should be a wee bit lavish in their ways, and should take the opportunity of turning round upon the Local Government Board and saying, "See what you've done? Look what you've let the poor ratepayers in for!"

### What is Efficient Vaccination?

A question which is attracting much attention at the present time is, what is efficient vaccination? The government standard, as everyone knows, is a purely arbitrary one, arrived at by methods of statistical computation which can never be devoid of chances of error. It is a good substantial standard for primary vaccination, and one that has stood the test of time; but what people are troubling about at present is not primary vaccination so much as the question, what is the minimum of secondary vaccination which will give protection for the time being? The frequency with which small-pox occurs among those who are reported to have been vaccinated has raised a strong feeling that in the presence of epidemic small-pox the only safety lies in recent re-vaccination. Hence it is now being asked, not what is efficient as a life-long protection, but what is the minimum which will tide one over present needs? And, unfortunately, this is not an easy question to answer. The criteria of efficiency are very unsatisfactory. It may indeed be doubted whether we know what is the exact relationship between the vesicle and the disease. At any rate it is evident enough that the fact of vaccination "taking" is not necessarily a proof that the patient was susceptible to small-pox. It would even seem that different brands of vaccine are not entirely protective against each other, as was shown by a case, mentioned at the last meeting of the Royal Medical and Chirurgical Society, in which three successful vaccinations were performed on a child in the course of four months. There are two ways in which a nation may protect itself from small-pox. One is universal vaccination, followed by a single re-vaccination, a method which England has refused to accept. The other is widespread re-vaccination whenever the disease shows itself. This "policy of scare" is apparently what we are drifting to, and when people now ask what is "efficient vaccination" they often mean what is "sufficient for the day." To this the Government "four mark" standard is no answer. The whole problem requires to be re-investigated.

